# The impact of family physician supply on district health system performance, clinical processes and clinical outcomes in the Western Cape Province, South Africa (2011–2014)

**DOI:** 10.4102/phcfm.v10i1.1442

**Published:** 2018-04-19

**Authors:** Rekai L. Chinhoyi, Moleen Zunza, Klaus B. von Pressentin

**Affiliations:** 1 Division of Epidemiology and Biostatistics, Stellenbosch University, South Africa; 2 Division of Family Medicine and Primary Care, Stellenbosch University, South Africa

## Abstract

**Background:**

A revised family physician (FP) training programme was introduced in South Africa in 2007. A baseline assessment (2011) of the impact of FP supply on district health system performance was performed within the Western Cape Province, South Africa. The impact of an increased FP supply within this province required re-evaluation.

**Aim:**

To assess the impact of FP supply on indicators of district health system performance, clinical processes and clinical outcomes in the Western Cape Province. The objectives were to determine the impact of FPs, nurses, medical officers (MOs) and other specialists.

**Setting:**

The study sample included all five rural districts and eight urban subdistricts of the Western Cape Province.

**Methods:**

A secondary analysis was performed on routinely collected data from the Western Cape Department of Health from 01 March 2011 until 30 April 2014.

**Results:**

The FP supply did not significantly impact the indicators analysed. The supply of nurses and MOs had an impact on some of the indicators analysed.

**Conclusion:**

This study did not replicate the positive associations between an increase in FP supply and improved health indicators, as described previously for high-income country settings. The impact of FP supply on clinical processes, health system performance and outcome indicators in the Western Cape Province was not statistically significant. Future re-evaluation is recommended to allow for more time and an increase in FP supply.

## Introduction

The World Health Organization (WHO) defines primary health care (PHC) as ‘essential health care’ that is based on scientifically sound and socially acceptable methods and technology. Primary health care is an aspect of health care that facilitates population access to health services through universal health care coverage of individuals, families and communities.^1^

From a global perspective, Africa has the least developed PHC systems, lowest life expectancy and the lowest numbers of doctors, nurses and midwives per capita.^2^ The South African PHC system is not exempt from these challenges as it is besieged by a quadruple burden of disease consisting of human immunodeficiency virus (HIV) or acquired immune deficiency syndrome (AIDS), tuberculosis (TB), non-communicable diseases (NCDs), high maternal and child mortality rates and high levels of violence and injuries.^3^

Shi et al. found that populations residing in regions with well-developed PHC systems have better health outcomes, lower health care costs, less specialist involvement per episode of care, more visits with primary care physicians and fewer hospital days in intensive care compared to regions with weak PHC systems.^4^

Family medicine is a comprehensive specialty that strives to ensure equitable access, continuity of care, coordination of care and comprehensiveness of care in PHC and district health services (DHS). To improve PHC, the principles of family medicine and primary care should be embraced by the entire PHC team,^5^ which includes clinical nurse practitioners, professional nurses, midwives, community health workers, doctors without postgraduate training in family medicine and allied health professionals. The main drivers of PHC are nurses with consultant support from doctors or family physicians (FPs).

The WHO defines FPs as ‘physicians who have specialized in the discipline of family medicine or general practice’.^6^ In South Africa, the roles of FPs in PHC settings and the DHS were defined as providing clinical care; acting as a consultant to the clinical team; providing mentoring and capacitating roles to other clinical staff; leading clinical governance at facility and subdistrict level; supervising students, interns or registrars and supporting community-based services, such as ward-based outreach teams.^7^

Although the positive impact of FPs on PHC is recognised globally, the impact of FPs within the DHS of the Western Cape (and South Africa) remains to be established. Since the discipline’s accreditation as a new specialty in 2007, the South African academic family medicine departments have revised their training programmes to produce FPs equipped to work independently in PHC facilities and district hospitals.^8^ Initial qualitative assessments of the impact of FP supply in the Western Cape indicate that FPs are improving clinical processes for serious and chronic conditions such as HIV infection, TB, NCDs and childhood diarrhoea.^5^ Since 2007, the FP supply has increased across the nine programmes taught nationally, albeit lower than the projected numbers of 70 new FPs per year. To date, only 18–20 graduates a year qualify nationally.^9^

**FIGURE 1:**
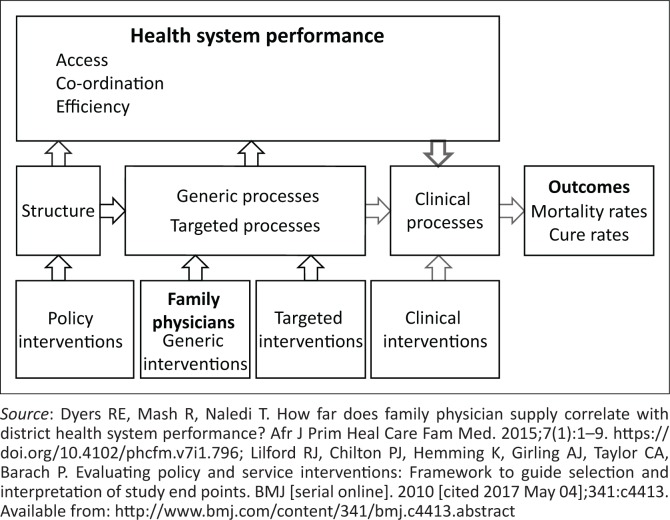
Modified Donabedian causal chain as conceptual framework.^8,11^

In a Western Cape pilot study by Dyers et al.,^8^ which examined data on health system performance between 01 April 2011 and 31 March 2012, it was concluded that strong correlations between FP supply and health system performance could not be demonstrated at that particular time as the supply of FPs was still negligible to make a demonstrable impact on health system performance in terms of access, coordination of care and efficiency; quality of clinical processes; as well as the odds of achieving favourable clinical outcomes (as depicted by the modified Donabedian causal chain, Dyers et al., Figure 1).^8^ Low FP numbers may result in PHC inequities for the communities dependent on public sector health services.^10^

In the last 4 years (2011–2014), the supply of FPs in Western Cape district hospitals and primary care facilities has doubled, from 22 to 45.^12^ This provides an opportunity to re-evaluate the impact of FPs on health indicators, using the methods and conceptual framework piloted by Dyers et al.^8^

The aim of this study was to determine the impact of FP supply on indicators of health system performance, clinical processes and outcomes. The primary objective was to determine the impact of FPs on designated health indicators, whilst the secondary objective was to determine the impact of other health care worker categories working within the DHS on these indicators, specifically nurses, medical officers (MOs) and general specialists.

## Methods

### Study design

The researchers conducted a population-based study by conducting secondary analyses of routinely collected data sourced from the Western Cape Department of Health’s District Health Information System (DHIS), for the time period from 01 March 2011 until 30 April 2014.

### Study population and sampling strategy

The study sample included all five rural districts and eight urban subdistricts of the Western Cape. Together these districts and subdistricts made up the 13 units of analysis. Analysis was performed at district level because FP supply was infused at a district or subdistrict level. Therefore, districts were the appropriate units of analysis that permitted the varied analysis of the impact of FP supply on district health indicators from 2011 to 2014.

### Study setting

**FIGURE 2:**
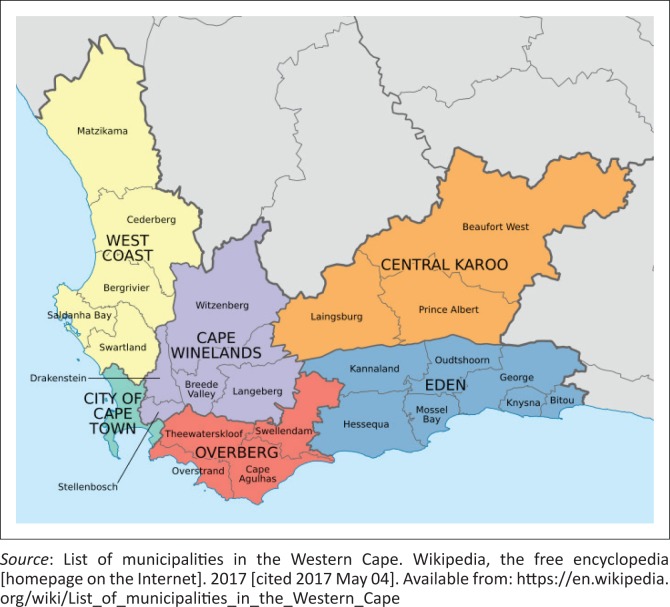
Map of the Western Cape Province and its district municipalities.^13^

The Western Cape consists of five rural districts (Eden, Central Karoo, Cape Winelands, Overberg and the West Coast district), as well as the Cape Town Metropole (City of Cape Town), the only urban district in the province (Figure 2). The metropole is divided into four sub-structures, which are, in turn, further divided into eight subdistricts. Therefore, there are eight sub-districts within the metropole including Western, Southern, Northern, Eastern, Khayelitsha, Klipfontein, Mitchells Plain and Tygerberg subdistricts. The Western Cape Province’s public health sector consists of 1326 health care facilities, which include 66 Community Health Centres, 267 Community Day Centres, 305 clinics, 34 district hospitals, 5 regional hospitals, 1 tertiary hospital, 6 TB treatment facilities and 4 psychiatry facilities.^14^ According to the District Health Barometer 2014–2015, 83% of the total provincial population (6 130 791 people) were dependent on the services provided by these public health sector facilities (Table 1).^15,16^ Table 1 shows the dependent and total populations in each of these areas for the same period.

**TABLE 1:** Description of units of analysis: dependent population (total population).

Unit of analysis	2011	2012	2013	2014
Western Cape Town Subdistrict	353 796 **(464 849)**	360 771 **(473 515)**	367 814 **(482 252)**	374 936 **(491 076)**
Southern Cape Town Subdistrict	427 482 **(561 664)**	436 692 **(573 162)**	445 984 **(584 743)**	455 366 **(596 419)**
Northern Cape Town Subdistrict	283 094 **(371 953)**	289 128 **(379 482)**	295 207 **(387 055)**	301 333 **(394 673)**
Eastern Cape Town Subdistrict	385 479 **(506 476)**	392 845 **(515 612)**	400 224 **(524 746)**	407 623 **(533 887)**
Khayelitsha Subdistrict	321 136 **(421 936)**	326 260 **(428 219)**	331 368 **(434 467)**	336 470 **(440 694)**
Klipfontein Subdistrict	289 425 **(380 272)**	295 134 **(387 366)**	300 872 **(394 483)**	306 641 **(401 626)**
Mitchells Plain Subdistrict	319 810 **(420 194)**	325 377 **(427 060)**	330 930 **(433 893)**	336 480 **(440 707)**
Tygerberg Subdistrict	453 939 **(596 425)**	462 985 **(607 672)**	472 073 **(618 949)**	481 205 **(630 262)**
Eden District	467 834 **(566 752)**	476 874 **(576 277)**	485 993 **(585 832)**	495 190 **(595 542)**
Central Karoo District	49 541 **(69 235)**	50 498 **(70 218)**	51 464 **(71 231)**	52 438 **(72 267)**
Cape Winelands District	552 374 **(772 249)**	563 048 **(789 963)**	573 815 **(808 041)**	584 674 **(826 439)**
Overberg District	184 184 **(259 652)**	187 743 **(266 109)**	191 334 **(272 624)**	194 955 **(279 189)**
West Coast District	244 251 **(400 438)**	248 970 **(409 411)**	253 732 **(418 608)**	258 533 **(428 012)**
**Total**	4 332 345 (**5 792 095)**	4 416 325 (**5 904 066)**	4 500 810 **(6 016 924)**	4 585 844 **(6 130 793)**

The figures in bold represent total population whilst the numbers not in bold represent the dependent population.

## Eligibility criteria

### Inclusion criteria

Data from Western Cape DHS facilities (clinics, community day centres, community health centres, dental clinics, district hospitals, health posts, midwife obstetrics units, mobile services, reproductive health services and satellite clinics) were included in the study.

### Exclusion criteria

Data from correctional services, environmental health offices, health promotion services, home-based care and non-medical sites were excluded. Community-based services, whilst part of DHS, were excluded because of different procedures in data collection and resource allocation between the units of analysis in this study.^8^

#### Data collection

Data were obtained from the Western Cape Department of Health’s routinely collected database using the formal data application process. The data were routinely collected from PHC and DHS facilities in all 13 units of analysis. Health facilities submit monthly data on agreed indicators into the DHIS, as determined by an information management protocol. The DHIS data are aggregated in a sequential manner at subdistrict, district, provincial and, ultimately, at national level.

Data were extracted from several aspects of the DHIS: Persal (human resources database), Sinjani (database of aggregated performance data of hospitals), ETR.net (electronic TB registry) and the National Population Census 2011. Data on the health system performance indicators were limited to PHC utilisation only (and not on the other domains described in the conceptual framework, Figure 1). Human resource data were obtained from the human resources database, whilst PHC utilisation, clinical outcome and clinical process data were derived from the Sinjani database on a variety of performance aggregated data from district hospitals and the electronic TB registry. Population data were derived from the National Population Census 2011.^17^ Data were linked using district and subdistrict names. Data from outside the public health sector and district health system, such as private, central, specialised and regional hospitals, were excluded.

Dependent variables or indicators were chosen for this study based on the Donabedian conceptual framework (Figure 1), the availability within the routinely collected data set provided by the Western Cape Department of Health and the likelihood of FPs having an impact on them (as predetermined by the pilot study).^8^

The variables were selected from the health system performance, clinical processes and clinical outcome domains of the Donabedian causal chain (see Table 2).^8^ Primary health care utilisation was used to assess health system performance. Cervical smears, TB treatment, couple year protection rate, early antenatal booking and under 1 year immunisation coverage were the designated clinical processes indicators, whilst maternal mortality, perinatal mortality and under-5 mortality were the indicators used to assess clinical outcomes. Dependent variables were calculated according to the Western Cape Department of Health’s Annual Performance Plan 2016–2017 definitions (Table 2) and formulas (Table 2-A1).^18^

**TABLE 2:** Description of dependent variables.

No.	Dependent variables	Definitions
1	PHC utilisation (access: health system performance)	Rate at which PHC services are utilised by the target population, represented as the average number of visits per person during the reporting period in the target population.^18^
2	Cervical smears (clinical processes)	Cervical smears (clinical process): Proportion of women aged 30 years and older who have screening for cervical cancer.^18^
3	TB treatment (clinical processes)	Proportion of patients suspected of having TB who have started treatment.^18^
4	Isoniazid preventive therapy (IPT) (clinical processes)	TB preventive therapy for HIV-positive people newly enrolled in HIV care (percentage).
5	CYPR (clinical processes)	Couple year protection rate: percentage women of reproductive age (15–44 years) who are using (or whose partner is using) a modern contraceptive method.
6	Early antenatal booking (clinical processes)	Percentage of pregnant women who visit a health facility for the primary purpose of receiving antenatal care, often referred to as ‘a booking visit’, which occurs before 20 weeks after conception.^18^
7	Immunisation coverage (clinical processes)	Percentage of all children under 1 year who complete their primary course of immunisation; a primary course includes BCG, OPV 0 & 1, DTaP-IPV-Hib 1, 2 & 3, Hep B 1, 2 & 3 and first measles at 9 months.
8	Maternal mortality (clinical outcomes)	Number of maternal deaths in facility expressed per 100 000 live births; a maternal death is the death of a woman whilst pregnant or within 42 days of termination of pregnancy, irrespective of the duration and the site of the pregnancy, from any cause related to or aggravated by the pregnancy or its management, but not from accidental or incidental causes.^18^
9	Perinatal mortality (clinical outcomes)	Stillbirths plus the number of children who have died in a health facility between birth and 28 days of life, expressed per 1000 total births in facility.^18^
10	Under-5 mortality (clinical outcomes)	The number of children who have died in a health facility between birth and their fifth birthday, expressed per 1000 live births in facility.^18^

PHC, primary health care; TB, tuberculosis; CYPR, couple year protection rate; BCG, Bacillus Calmette–Guérin; OPV, oral polio vaccine; DTap, diphtheria, tetanus and pertussis; Hep B, hepatitis B; IPV, inactivated polio vaccine; Hib, haemophilus influenzae type b; IPT, isoniazid preventive therapy.

#### Data analysis

The impact of FPs and other health care worker categories on health system performance, clinical processes and outcomes was measured through statistical measures of association. The primary predictor variable for this study was FPs employed within the DHS per 10 000 dependent population (a generic intervention according to the conceptual framework). The data on FP supply were obtained via the academic departments of family medicine at the Western Cape training institutions.^12^ The other predictor variables, such as nurse, MO and specialist supplies, were included because of their potential confounding effect on health system performance, clinical processes and outcomes.^19^ The general specialists category excluded FPs (see Table 3 for a description of the predictor variables).

**TABLE 3:** Description of predictor variables.

Predictor variable	Definition
Supply of family physicians	Family physicians per 10 000 dependent population
Supply of nurses	Nurses per 10 000 dependent population
Supply of medical officers	Medical officers per 10 000 dependent population
Supply of specialists	Specialists employed by district health services, other than family physicians, per 10 000 dependent population

These include specialists working in the district health services, such as internal medicine, paediatrics, obstetrics and gynaecology and anaesthetics. In the Western Cape, these specialists are not involved in the District Clinical Specialist Team model used in the other South African provinces.

Analysis between each predictor variable (FPs, nurse, MO and specialist supply) and each rate outcome (PHC utilisation, under-5 mortality, maternal mortality, perinatal mortality and couple year protection rates) was performed using generalised linear models with a negative binomial link (log), and summarised using incident rate ratios (IRRs) with corresponding 95% confidence intervals. Analysis between each predictor variable (FPs, nurse, MO and specialist supply) and each proportional outcome (cervical smears, TB treatment and early antenatal booking) was performed using a binomial link (logit) and summarised as odds ratios (OR) with corresponding 95% confidence intervals. All analyses were performed using the statistical software package STATA 13.^20^

### Ethical considerations

The study was approved by the Human Research Ethics Committee of Stellenbosch University (N11/10/012).

## Results

All 13 districts and subdistricts of the Western Cape were included as the units of analysis for this study. The total populations and dependent populations of these districts are described in Table 1. The full-time equivalent distribution of FPs and other health care categories per district or subdistrict is summarised in the appendix as Table 2-A1. Bivariate analysis was used to assess the impact of time on each health care indicator or outcome (2011–2014) and to adjust for within district or subdistrict clustering (Table 4). Outcome variables that significantly changed over the reporting period (*p* < 0.1) underwent further bivariate analysis with each predictor variable whilst the effects of time were held constant (Table 5).

**TABLE 4:** Bivariate analysis of the impact of time on each outcome variable (2011–2014).

Outcome	Mean	s.d.	Bivariate analysis IRR/OR (95% CI)	*p*
PHC utilisation (health system performance)	2.500	0.560	IRR 0.94 (0.922–0.97)	0.0001*
Cervical smears (clinical processes)	0.560	0.170	OR 0.97 ( 0.88–1.06)	0.5000
TB treatment (clinical processes)	0.630	0.360	OR 0.24 (0.19–0.31)	0.0001*
Isoniazid preventive therapy (IPT) (clinical processes)	0.215	0.269	OR 2.70 (1.92–3.79)	0.0001*
Couple year protection rate (CYPR) (clinical processes)	0.530	0.140	IRR 0.94 (0.87–1.02)	0.1100
Early antenatal booking (clinical processes)	0.610	0.090	OR 0.25 (0.13–0.37)	0.0900*
Immunisation coverage (clinical processes)	0.810	0.150	OR 0.94 (0.79–1.12)	0.4800
Maternal mortality (clinical outcomes)	13.920	20.620	IRR 1.28 (0.81–2.02)	0.2800
Perinatal mortality (clinical outcomes)	14.890	8.590	IRR 0.99 (0.96–1.02)	0.5500
Under-5 mortality (clinical outcomes)	5.820	5.180	IRR 1.16 (1.03–1.31)	0.0200*

Outcome variables that changed over the 4-year reporting period (*, *p* < 0.1 significance level).

IRR, incident rate ratio; PHC, primary health care; s.d., standard deviation; OR, odds ratio; TB, tuberculosis.

**TABLE 5:** Bivariate analysis between each predictor variable and outcome.

Outcomes	Health care worker	Mean	s.d.	Bivariate analysis IRR (95% CI)	*p*
**Rate outcomes**					
PHC utilisation (access)	Family physician	2.500	0.560	IRR 1.24 (0.34–4.51)	0.743
	Nurses	2.500	0.560	IRR 1.01 (1.0–1.02)	0.080*
	Medical officers	2.500	0.560	IRR 1.18 (1.09–1.27)	0.001*
	Specialist	2.500	0.560	IRR 0.99 (0.52–1.90)	0.990
Under-5 mortality rate (clinical outcomes)	Family physician	5.820	5.180	IRR 2.74 (0.001–9364.24)	0.810
	Nurses	5.820	5.180	IRR 1.09 (1.05–1.14)	0.001*
	Medical officers	5.820	5.180	IRR 1.35 (0.62–2.95)	0.450
	Specialist	5.820	5.180	IRR 0.09 (0.01–0.99)	0.049*
**Proportional outcomes**					
Early antenatal visit (clinical processes)	Family physician	0.610	0.090	OR 8.75 (0.37–205.39)	0.178
	Nurses	0.610	0.090	OR 1.02 (1.01–1.03)	0.005*
	Medical officers	0.610	0.090	OR 0.83 (0.65–1.05)	0.110
	Specialist	0.610	0.090	OR 0.53 (0.29–0.99)	0.047*
TB treatment (clinical processes)	Family physician	0.630	0.360	OR 2.74 (0.12–61.32)	0.530
	Nurses	0.630	0.360	OR 1.00 (0.97–1.04)	0.680
	Medical officers	0.630	0.360	OR 1.09 (0.75–1.58)	0.660
	Specialist	0.630	0.360	OR1.19 (0.16–8.86)	0.870
IPT (clinical processes)	Family physician	0.215	0.269	OR 20.22 (0.0006–653 639)	0.570
	Nurses	0.215	0.269	OR 1.04 (1.01–1.07)	0.011
	Medical officers	0.215	0.269	OR 0.86 (0.47–1.54)	0.600
	Specialist	0.215	0.269	OR 0.21 (0.01–7.44)	0.390

Health indicators or outcomes that were significantly impacted by health care worker categories (*, *p* < 0.1 significance level).

IRR, incident rate ratio; PHC, primary health care; s.d., standard deviation; IPT, isoniazid preventive therapy; OR, odds ratio; TB, tuberculosis.

The following outcomes did not change significantly between 2011 and 2014: cervical screening, OR 0.97 (95% CI 0.88–1.06, *p* = 0.49), couple year protection rate, IRR 0.94 (95% CI 0.87–1.02, *p* = 0.11), immunisation coverage, OR 0.94 (95% CI 0.79–1.12, *p* = 0.47), maternal mortality, IRR 1.28 (95% CI 0.81–2.02, *p* = 0.28) and perinatal mortality, IRR 0.99 (95% CI 0.96–1.02, *p* = 0.55) (Table 4). Hence, no further analysis was conducted on these outcome variables. The results of the bivariate analysis between predictor variables (health care worker categories) and outcome variables that had changed over the 4-year reporting period are summarised in Table 5.

Associations between predictor variables and outcome variables that exhibited significant associations of *p* < 0.1 underwent further multivariate analysis (Table 6).

**TABLE 6:** Multivariate analysis between each predictor variable and outcome variables that had a significant association (*p* < 0.1) in the bivariate analysis.

Outcomes	Health care worker	Mean	s.d.	Bivariate analysis IRR(95% CI)	*p*
**Rate outcomes**					
PHC utilisation (access)	Medical officers	2.500	0.560	IRR 1.26 (1.12–1.41)	0.0010*
Under-5 mortality rate (clinical outcomes)	Nurses	5.820	5.180	IRR 1.14 (1.07–1.21)	0.0020*
**Proportional outcomes**					
Early antenatal visit (clinical processes)	Nurses	0.610	0.090	OR 1.02 (1.01–1.03)	0.0001*
IPT (clinical processes)	Nurses	0.215	0.269	OR 1.09 (1.031.17)	0.0070

Health indicators or outcomes that were significantly impacted by health care worker categories (*, *p* < 0.1 significance level).

IRR, incident rate ratio; PHC, primary health care; s.d., standard deviation; IPT, isoniazid preventive therapy; OR, odds ratio; TB, tuberculosis.

Family physician supply and specialist supply had no significant impact on PHC utilisation. However, nurse supply was associated with a 1% increase in PHC utilisation, IRR 1.01 (95% CI 1.0–1.02, *p* = 0.08), whilst MO supply was associated with an 18% increase in PHC utilisation, IRR 1.18 (95% CI 1.09–1.27, *p* = 0.001) after adjusting for the effects of time. Multivariate analysis of nurse supply against PHC utilisation revealed that there was no significant association between nurse supply and PHC utilisation: IRR 0.99 (95% CI 0.98–1.00, *p* = 0.304) after adjusting for the effects of other health care worker categories. MO supply was significantly associated with PHC utilisation in both bivariate and multivariate analysis: IRR 1.26 (95% CI 1.12–1.41, *p* = 0.0001) (Table 6).

Family physician supply and MO supply were not significantly associated with the incidence of under-5 mortality. Nurse supply was associated with a 9% increase in the incidence of under-5 mortality, whilst specialist supply was associated with a 91% decrease in the incidence of under-5 mortality rate. Multivariate analysis in which the impacts of other health care workers were held constant revealed that nurse supply was associated with a 14% increase in the incidence of under-5 mortality: IRR 1.14 (95% CI 1.07–1.21, *p* = 0.002) (Table 6). It further revealed that specialist supply had no statistically significant association with under-5 mortality: IRR 0.38 (95% CI 0.05–3.15, *p* = 0.37).

Family physician supply and MO supply had no significant impact on early antenatal visits. Nurse supply was associated with a 2% increase in the odds of early antenatal visits: OR 1.02 (95% CI 1.01–1.03, *p* = 0.005). Specialist supply was associated with a 47% decrease in the odds of early antenatal visits OR 0.53 (95% CI 0.29–0.99, *p* = 0.05). Multivariate analysis indicated that nurse supply was associated with a 2% increase in the odds of early antenatal booking, OR 1.02 (95% CI 1.01–1.03, *p* = 0.0001) (Table 6), whilst it indicated that specialist supply had no significant impact on early antenatal visits: OR 0.91 (95% CI 0.68–1.22, *p* = 0.526).

Even though the proportion of patients on TB treatment varied over the reporting period, bivariate analysis revealed that none of the health care worker supplies had a significant impact on TB treatment.

Nurse supply was the only health care worker category to be associated with IPT, OR 1.04 (95% CI 1.01–1.07, *p* = 0.01). Multivariate analysis revealed that nurse supply was significantly associated with a 9% increase in IPT: OR 1.09 (95% CI 1.03–1.17, *p* = 0.007) (Table 6). No other health care worker category had an impact on this variable.

We did not perform any analysis by district, because there were only four data points recorded for each dependent variable (2011, 2012, 2013 and 2014); therefore, no meaningful analysis could be performed as we were not able to replicate the methods described by Dyers et al.^8^

## Discussion

The primary objective of this study was to determine the impact of FPs on designated PHC indicators in the district health system of the Western Cape. Bivariate analysis of the impact of FPs, the primary objective, indicated that FPs did not have a significant impact on the indicators assessed in this study. This may be explained by the fact that the FP supply is still too low to have made a significant impact on these health indicators, even though it has doubled over the past 4 years.^12^

However, bivariate analysis of secondary objectives revealed that increased nurse supply was associated with a 1% increase in PHC utilisation, IRR 1.01 (95% CI 1.0–1.02, *p* = 0.08), and a 2% increase in early antenatal bookings. This makes sense as nursing staff are the key providers of comprehensive antenatal care health services. In addition, nurse supply was associated with a 9% increase in IPT; this is also understandable as nurses are key clinical members of the vertical TB and HIV programmes, and the advocates for increased adherence to IPT.^21^ Nurse supply was also associated with a 14% increase in the incidence of under-5 mortality. This was an unexpected clinical outcome finding. This may be explained by inconsistent data as described in the 2014 Stats-SA Live Birth report, which highlighted an under-registration of births during 2013, resulting in a false increase in under-5 mortality rate. The Western Cape Department of Health is aware of these data discrepancies and measures are implemented to improve data accuracy.^18^

Medical officer supply was associated with an 18% increase in PHC utilisation: this may indicate greater reliance on the public health system by the population served in those facilities with MOs as part of the primary team; conversely, it could also point to the disease burden of these communities in which there was an increased supply at the PHC facilities (more MOs may be appointed at busier PHC facilities). Medical officers had no significant impact on early antenatal bookings. This is an unexpected finding as an increased supply of primary care doctors should be associated with improved maternal health care. However, this dependent variable is not so much an indication of care quality, but rather an indication of how pregnant women make use of the available health services. A number of unrelated factors or unmeasured confounders might explain why pregnant women choose to book early in their pregnancy. It may be that primary care facilities with more MOs are dealing with a more dependent community with greater socio-economic challenges, which negatively impact the uptake of available services. Conversely, MO supply was associated with a 47% reduction in under-5 mortality, which is more in keeping with the published benefits of an increased supply of primary care doctors.^22^ This perceived benefit may also be explained by other factors or confounders, such as targeted initiatives like maternal and child health policies and interventions.

Specialist supply was found to be significantly associated with a 91% decrease in the incidence of under-5 mortality rate. This was an unexpected clinical outcome finding and may be explained by inconsistent data as described in the 2014 Stats-SA Live Birth report.^18^ The health effects of expanding specialist supply are unknown, but expanding specialist supply would lead to increased costs, possibly with no proportional benefits for the health of the population.^23^ In primary care-oriented systems, the population dependent on PHC services have fewer specialists involved in an episode of care and more visits with primary care physicians, spend fewer hospital days in intensive care and have lower health care costs.^24^ The DHS is also an environment with a low supply of specialists, compared to other health worker cadres (available in Table 1-A1). Specialists have a direct and indirect influence on the care provided to patients: they function as consultants to the clinical team and have to exert their influence through other team members who provide care or organise services.^5^

## Study strengths

The strengths of this study included finite sampling which ensured that all Western Cape districts were included and represented in the study, thereby reducing the potential for selection bias.^25^ The high response rate (100%) ensured that sampling had no impact on the validity of the inferences made. Moreover, the study procedures, inclusion and exclusion criteria were uniform across districts and sub-districts, thereby reducing systematic selection bias or differential misclassification of districts. Furthermore, the study outcomes were clearly defined and objectively measured using data from DHIS. The use of generalised linear models using multivariate binomial and negative binomial regression ensured that potential confounders such as the supply of other health care categories (i.e. nurses, MOs and specialists) were adjusted for during the analysis.

## Study limitations

Because of the fact that there were only four data points measured for the dependent variables in the available data set, no district by district analysis could be performed. Furthermore, some outcomes specified in the original study protocol and baseline study by Dyers et al., such as TB cure, diabetes score, hypertension score, hospital expenditure and chronic care team coordination, could not be measured.^8^ The cause-and-effect relationship between FP supply and DHS could not be determined in terms of temporality.

## Recommendations

A follow-up study at 10 years or more when the newly trained FPs have matured in their role and their numbers have increased is recommended.^26^ In addition, a nationwide study including more districts and more FPs may increase the sample size and thereby increase the power of the study.

## Conclusion

This study did not replicate the positive associations between an increase in FP supply and improved health indicators, as described by Starfield and others in studies conducted across 19 countries, which revealed that a one unit increase in primary care physician supply resulted in improvements in all health outcomes.^26,27,28^ In this study, the supply of FP had no statistically significant impact on the indicators used to measure health system performance and clinical outcomes. This discrepancy may be attributed to the low supply and relatively shorter duration of FP supply within this South African province. The ongoing analysis of the impact of FPs on district health outcomes is recommended to allow for more time and an increase in FP supply.

## Appendix 1

**TABLE 1-A1:** Number of full-time staff equivalents per staff category in each district or subdistrict (2011–2014).

Study ID	District or Subdistrict	Setting	Time	FPs per 10 000 dp	Nurses per 10 000 dp	MOs per10 000 dp	Specialist per10 000 dp
1	Western CPT Subdistrict	1	2011	0.000	8.17	1.27	0.03
1	Western CPT Subdistrict	1	2012	0.000	6.43	1.17	0.03
1	Western CPT Subdistrict	1	2013	0.027	6.44	1.15	0.00
1	Western CPT Subdistrict	1	2014	0.027	6.61	1.22	0.06
2	Southern CPT Subdistrict	1	2011	0.023	11.95	1.52	0.41
2	Southern CPT Subdistrict	1	2012	0.023	13.03	1.53	0.37
2	Southern CPT Subdistrict	1	2013	0.045	13.43	1.52	0.39
2	Southern CPT Subdistrict	1	2014	0.044	13.24	1.60	0.45
3	Northern CPT Subdistrict	1	2011	0.035	2.72	0.45	0.00
3	Northern CPT Subdistrict	1	2012	0.035	2.73	0.49	0.00
3	Northern CPT Subdistrict	1	2013	0.034	2.71	0.50	0.00
3	Northern CPT Subdistrict	1	2014	0.033	2.72	0.53	0.00
4	Eastern CPT Subdistrict	1	2011	0.026	10.58	1.33	0.20
4	Eastern CPT Subdistrict	1	2012	0.025	10.64	1.29	0.19
4	Eastern CPT Subdistrict	1	2013	0.100	10.42	1.38	0.18
4	Eastern CPT Subdistrict	1	2014	0.147	10.55	1.37	0.21
5	Khayelitsha Subdistrict	1	2011	0.031	14.45	2.31	0.06
5	Khayelitsha Subdistrict	1	2012	0.061	17.29	3.26	0.10
5	Khayelitsha Subdistrict	1	2013	0.121	17.17	3.04	0.14
5	Khayelitsha Subdistrict	1	2014	0.119	17.15	2.81	0.25
6	Klipfontein Subdistrict	1	2011	0.104	17.31	2.19	0.49
6	Klipfontein Subdistrict	1	2012	0.102	16.53	2.20	0.48
6	Klipfontein Subdistrict	1	2013	0.100	7.94	0.97	0.00
6	Klipfontein Subdistrict	1	2014	0.098	7.66	1.02	0.00
7	Mitchel’s Plain Subdistrict	1	2011	0.063	7.69	0.87	0.00
7	Mitchel’s Plain Subdistrict	1	2012	0.061	7.71	0.97	0.04
7	Mitchel’s Plain Subdistrict	1	2013	0.060	14.90	2.51	0.59
7	Mitchel’s Plain Subdistrict	1	2014	0.059	16.82	2.70	0.09
8	Tygerberg Subdistrict	1	2011	0.022	13.61	1.72	0.21
8	Tygerberg Subdistrict	1	2012	0.065	13.84	1.72	0.19
8	Tygerberg Subdistrict	1	2013	0.064	14.40	1.78	0.12
8	Tygerberg Subdistrict	1	2014	0.062	14.75	1.98	0.20
9	Eden District	2	2011	0.021	15.39	0.87	0.08
9	Eden District	2	2012	0.042	15.37	1.19	0.11
9	Eden District	2	2013	0.062	15.39	1.17	0.06
9	Eden District	2	2014	0.081	15.49	1.12	0.08
10	Central Karoo District	2	2011	0.000	35.32	2.56	0.00
10	Central Karoo District	2	2012	0.000	35.05	1.96	0.00
10	Central Karoo District	2	2013	0.000	34.59	2.58	0.00
10	Central Karoo District	2	2014	0.000	32.23	2.67	0.19
11	Cape Winelands District	2	2011	0.063	14.57	1.06	0.16
11	Cape Winelands District	2	2012	0.080	14.46	1.04	0.14
11	Cape Winelands District	2	2013	0.096	14.41	1.04	0.05
11	Cape Winelands District	2	2014	0.094	14.45	1.07	0.02
12	Overberg District	2	2011	0.109	17.27	1.30	0.26
12	Overberg District	2	2012	0.213	16.99	1.30	0.32
12	Overberg District	2	2013	0.209	17.56	1.43	0.07
12	Overberg District	2	2014	0.205	17.75	1.44	0.11
13	West Coast District	2	2011	0.082	19.98	1.45	0.09
13	West Coast District	2	2012	0.080	19.92	1.72	0.11
13	West Coast District	2	2013	0.079	19.71	1.66	0.02
13	West Coast District	2	2014	0.116	19.73	1.77	0.06

Setting: 1 = urban (Cape); 2 = rural.

CPT, Cape Town; dp, dependent population; FPs, family physicians; MOs, medical officers.

**TABLE 2-A1:** Variable determination.

Type	Indicator	Calculation
Predictor variable	Family physician supply	$=\frac{\text{No. of family phisicians}}{\text{Dependent population}}\times 10\;000$
	Nurse supply	$=\frac{\text{No. of nurses}}{\text{Dependent population}}\times 10\;000$
	Medical officer supply	$=\frac{\text{No. of Medical Officers}}{\text{Dependent population}}\times 10\;000$
	Specialist supply	$=\frac{\text{No. of specialist}}{\text{Dependent population}}\times 10\;000$
Dependent variable	PHC utilisation	$\frac{\text{PHC headcount}}{\text{subdistrict or District}}$
	Cervical smears	$\frac{\text{Cervical cancer screening in woman }30\text{ years and older}}{\text{Female population }30\text{ years and older}÷10}$
	TB treatment	$\frac{\text{Suspected TB patients initiated on treatment}}{\text{Total suspected of TB}}$
	IPT in newly diagnosed HIV patients	$\frac{\text{No. of new HIV patients started on IPT}}{\text{No. of new HIV patients}}$
	CYPR	$\frac{\text{Contraceptive years equivalent}}{\text{Female population }15-44\text{ years}}$ Contraceptive years equivalent = Sum of: Male sterilisations × 20 Female sterilisations × 10 Medroxyprogesterone injection ÷ 4 Norethisterone enanthate injection ÷ 6 Oral pill cycles ÷ 13 IUCD inserted × 4 Male condoms ÷ 200 *The couple year protection rate was calculated by the DHIS NDoH5 method because there was no subdermal implants data recordings and no data on female condom use or distribution before 2015*
	Early antenatal booking	$\frac{\text{Antenatal first visist before }20\text{ weeks}}{\text{Total antenatal viists}}$ *(Total antenatal visits = sum of antenatal first visit before 20 weeks + antenatal first visit 20 weeks or later)*
	Immunisation coverage	$\frac{\text{Immunised fully under }1\text{ year}}{\text{Total population under }1\text{ year}}$
	Maternal mortality	$\frac{\text{Maternal death in facility}}{\text{Live births in facility}}\times 100\;000$
	Under-5 mortality	$\frac{\text{Children under }5\text{ years who died in facility}}{\text{Live births facility}}\times 100$
	Perinatal mortality rate	$\frac{\text{Still births and inpatient early neonatal deaths in facility}}{\text{Total births in facility}}\times 100$
